# A novel method to generate *Salmonella* Typhi Ty21a ghosts exploiting the λ phage holin-endolysin system

**DOI:** 10.18632/oncotarget.18383

**Published:** 2017-06-06

**Authors:** Gayeon Won, Boram Kim, John Hwa Lee

**Affiliations:** ^1^ College of Veterinary Medicine, Chonbuk National University, Iksan Campus, Iksan, Republic of Korea

**Keywords:** *Salmonella* Typhi, holin-endolysin system, bacterial ghosts, inactivated vaccine, typhoid fever

## Abstract

Human typhoid fever caused by *Salmonella* Typhi still poses a severe global disease burden in developing countries despite the availability of commercial vaccines. In this study, we constructed a non-living *S*. Typhi Ty21a vaccine candidate by employing a lambda (λ) phage-derived holin-endolysin system to efficiently construct bacterial ghosts. The lysis plasmid pJHL464 harbors an R lysis cassette that is stringently regulated by dual promoters containing *cI857/λPR and P_araBAD_/araC* components. The plasmid was introduced into an *asd* gene-deleted *S*. Typhi Ty21a strain designated JOL1675. The *in vitro* expression of endolysin (~17.76 kDa) in the subsequent JOL1675 vaccine construct when grown under lysis inducible conditions was validated by immunoblotting. In scanning electron microscopy analysis, surface transmembrane tunnels and a collapsed body were visualized in the ghosts. Following 48 h of lysis, no viable JOL1675 cells remained, indicating that lysis of all cells was achieved. Subcutaneous immunizations of mice with the JOL1675 ghosts produced significantly increasing titers of serum IgG and vaginal wash secretory IgA antibodies against JOL1675 outer membrane proteins during the observational period. Further, serum collected at 6 weeks post-immunization of rabbits exhibited effective bactericidal activity against wild type *S*. Typhi in the presence of complement. These data showed that JOL1675 ghosts are highly immunogenic and elicit humoral and mucosal responses expected to correlate with protective immunity against *S*. typhi. Collectively, our findings support the conclusion that incorporating a λ phage holin-endolysin-mediated lysis construct into *S*. Typhi is an efficient strategy for developing a novel and safe non-living typhoid vaccine candidate.

## INTRODUCTION

*Salmonella* enterica serovar Typhi (*S*. Typhi), a human-restricted pathogen, is the etiological agent of human typhoid fever, which is an important global health problem leading to approximately 20 million illnesses and 0.2 million deaths worldwide annually, particularly in immunocompromised individuals [1]. The emergence of antibiotic-resistant *S*. Typhi strains and low sensitivity of diagnostic tests have increased public health concerns [2]. Currently, two approved typhoid vaccines, a parenteral Vi capsular polysaccharide vaccine (Typherix® or Typhim Vi®) and an oral live attenuated Ty21a (Vivotif®), are available [3]. Despite the presence of these licensed vaccines and a high demand for vaccines in endemic areas, the vaccines are mostly offered to persons traveling from developed countries to affected countries rather than to the individuals residing in the endemic countries due to poor utilization of health care services [4, 5], resulting in disease eradication failure. A recent systemic review reported that Ty21a and the Vi polysaccharide vaccine confer on average 51% and 55% of moderate protective efficacy, respectively [5]. However, the licensed vaccines still have deficiencies. Neither vaccine can be administered to children younger than 2 years [5]. Further, failure of the Vi vaccine to elicit either mucosal immunity or a booster effect prompted development of a new vaccine consisting of Vi polysaccharide conjugated to recombinant *Pseudomonas aeruginosa* exotoxin A (rEPA), but the conjugated Vi polysaccharide vaccine is not widely available in developing countries [6]. Further, live attenuated Ty21a vaccines still carry the potential risk of unacceptable reactogenicity [7]. Thus, a new strategy to develop more optimal typhoid vaccine candidate has great potential value.

Bacterial ghosts generated by expressing the bacteriophage PhiX174 lysis gene *E* in gram-negative bacteria have the potential to function as a vaccine platform [8]. A hydrophobic E protein encoded by lysis gene *E* oligomerizes pore-forming channels between the inner and outer membranes of the cell envelope, through which cytoplasmic contents are expelled [8, 9]. The empty intact bacterial envelopes containing lipopolysaccharides, outer membrane proteins, peptidoglycans, and lipid A retain their cellular morphology and antigenic traits [9]. Due to their safety and potential to induce local immunity, such bacterial ghosts were prepared for human non-living vaccine candidates [10, 11]. However, the lysis gene *E* does not reliably kill all of the target bacteria and convert them to bacterial ghosts. In previous studies, roughly 1.2% of *Escherichia coli* cells remained viable during the ghost preparation process [12], and Chetan *et al*. reported that 3×10^3^ CFU of viable cells were recovered from *S*. Enteritidis ghosts prepared by use of protein E following 48 h of lysis [13]. In an effort to improve lysis efficiency and reduce safety concerns, we have used a holin-endolysin-mediated lytic mechanism originating from the large bacteriophage λ to develop a novel *S*. Typhi non-living vaccine candidate.

Two proteins, endolysin and holin, are instrumental for host bacterial lysis by the double-stranded DNA λ phage [14]. Endolysin is a peptidoglycan degrading enzyme that weakens the structural integrity of the peptidoglycan layer according to a programmed cell lysis mechanism regulated by holin, a small hydrophobic pore-forming protein. Accumulated endolysin molecules allow for cytoplasmic content release and initiate the degradation of the peptidoglycan layer when holin forms membrane lesions at a predetermined time [14]. The strong lytic activity of the holin-endolysin system renders these proteins as attractive candidates to develop antimicrobials [15]. In the present study, the potent lytic activity of holin-endolysin encoded by the λ phage *S* and *R* genes, respectively, was stringently governed by a convergent promoter system containing a sense λpR promoter with *cI_857_* and an anti-sense *P_araBAD_* promoter with the *araC* regulatory components [13]. We constructed an *asd*^+^ lysis plasmid (pJHL464) harboring an R lysis cassette consisting of the *S, R, Rz*, and *Rz1* genes placed between the dual promoter components. The lysis plasmid was introduced into an *asd*-deleted *S*. Typhi Ty21a vaccine strain to prepare a novel non-living vaccine candidate for typhoid fever. Further, the present study explored the potential immunogenicity of the vaccine candidate.

## RESULTS

### Characterization of a *S*. Typhi ghost constructed by expression of the R lysis cassette

**Figure 1:**
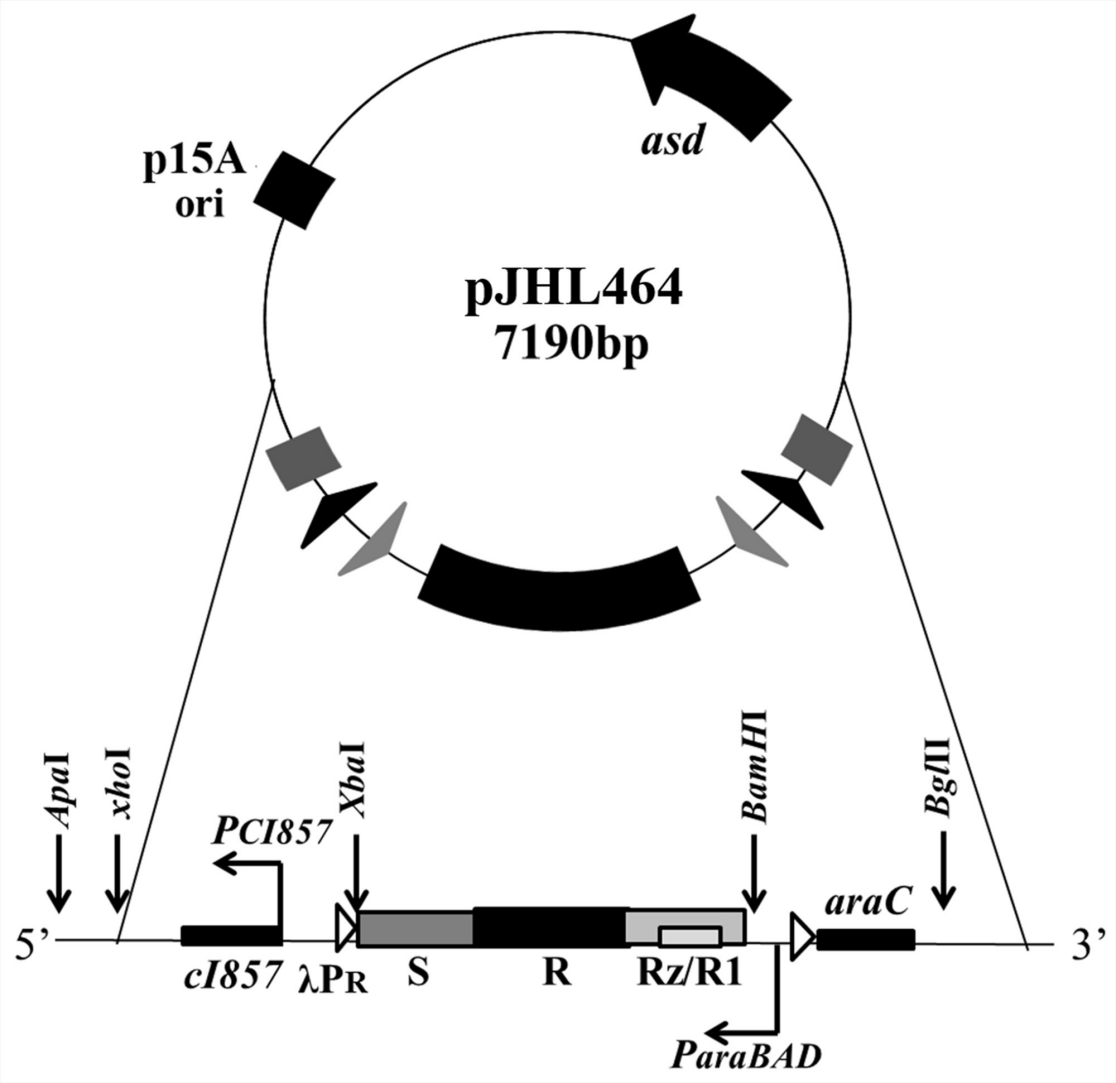
Components of the ghost plasmid pJHL464 The *asd*^+^ plasmid with orip15A carrying the constitutive expression system of the lysis genes regulated by the convergent promoter elements is shown.

**Figure 2:**
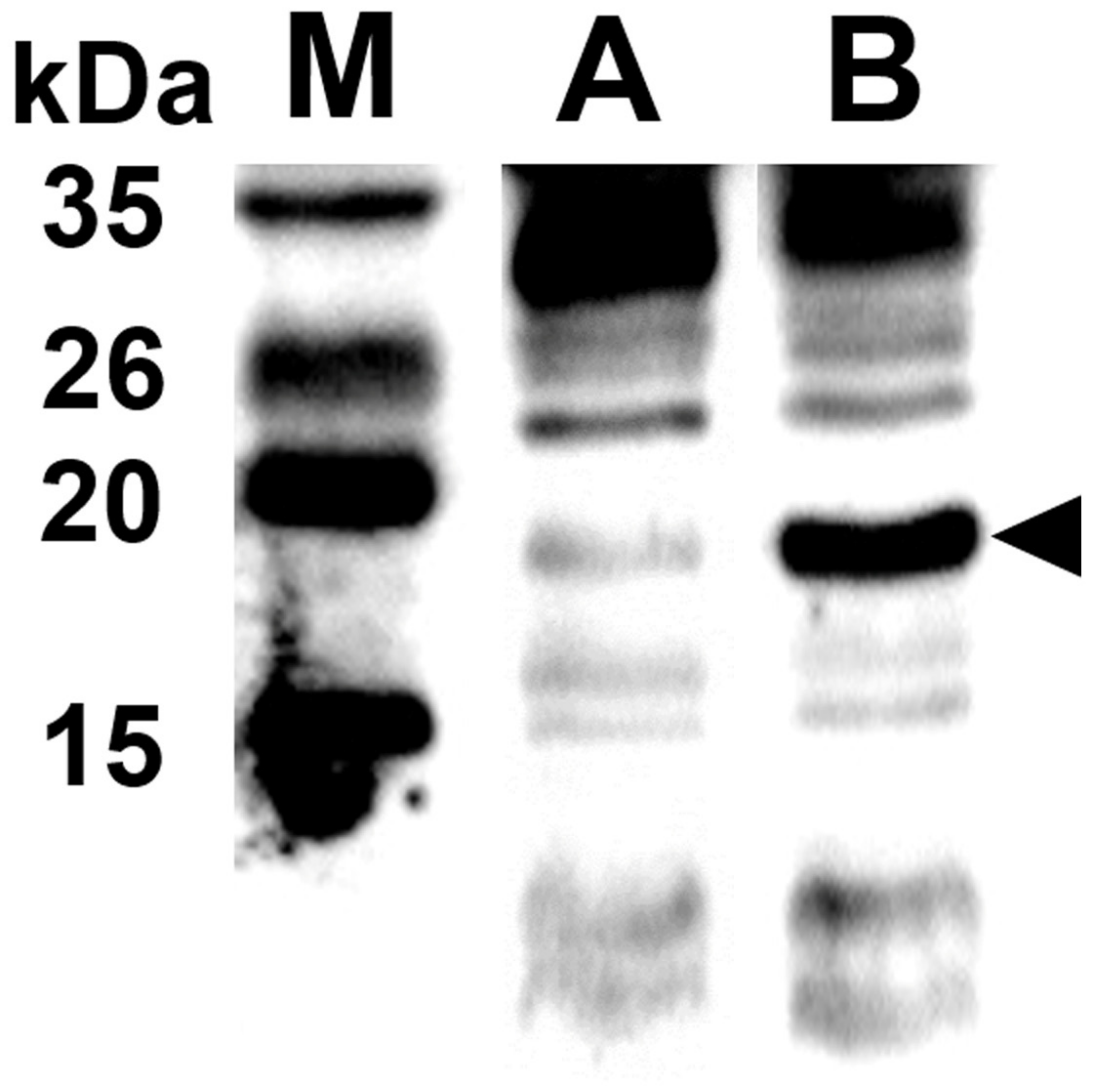
Endolysin and protein E expressed in JOL1675 confirmed by rabbit anti-endolysin polyclonal antibodies The black arrowhead indicates the expected size of endolysin protein. Lane M, size marker; lane A, JOL1675 grown in the presence of L-arabinose at 27°C; lane 2, JOL1675 ghosts produced under lysis conditions.

**Figure 3:**
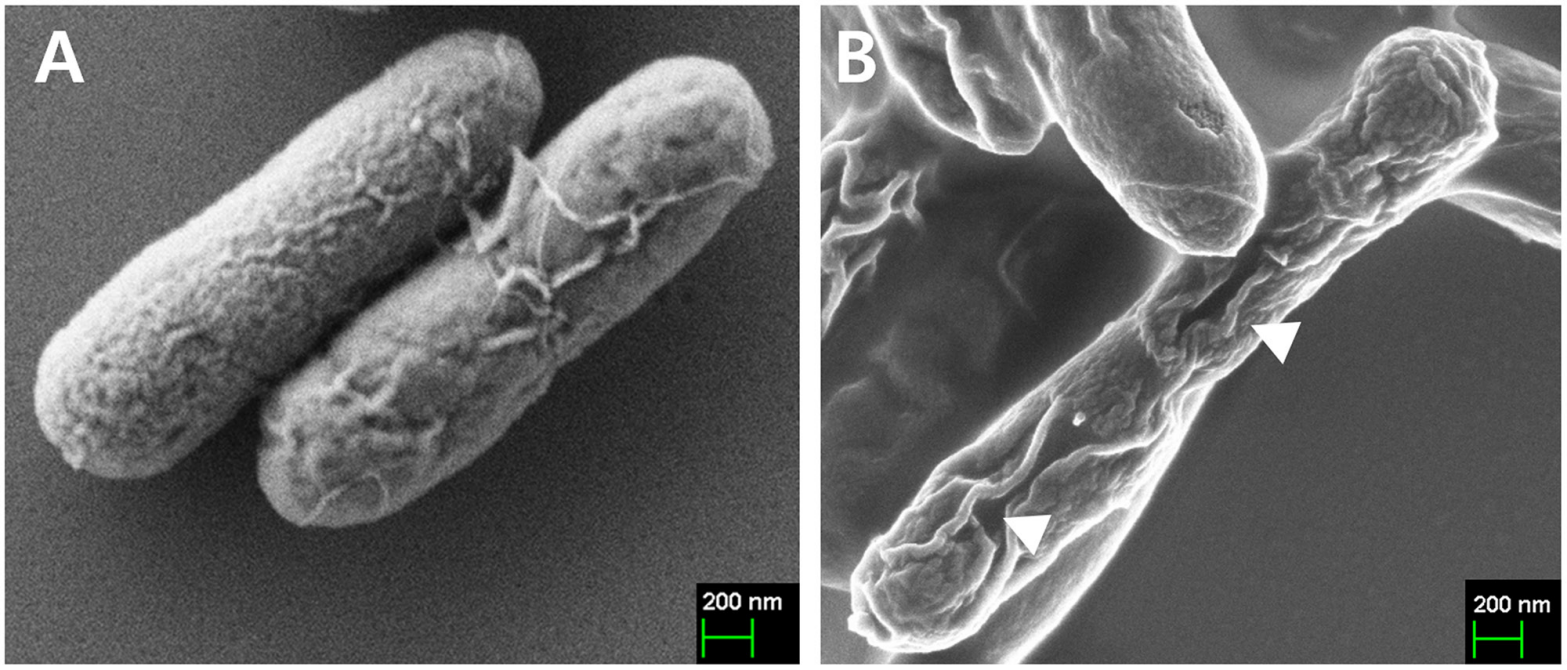
Characterization of JOL1675 *S*. Typhi ghost cells by scanning electron microscopy (SEM) analysis **(A)** Intact JOL1675 cells before lysis. **(B)** JOL1675 ghost cells after 24 h of lysis. The arrows indicate transmembrane tunnels.

The ghost plasmid pJHL464 constructed in this study consists of the *S*, *R*, and *Rz*, and *Rz1* genes transcriptionally inhibited in the presence of arabinose by the anti-sense arabinose inducible *P_araBAD_* promoter or transcriptionally activated by the sense thermo-sensitive λpR-*cI_857_* promoter (Figure 1). The expression of endolysin in JOL1675 harboring pJHL464 under optimal lysis conditions was confirmed by Western blot analysis. An immunoreactive band of ~17.7 kDa corresponding to the predicted molecular mass of the endolysin protein was detected in the pellet of JOL1675 ghosts incubated under optimal lysis conditions at 42°C in the absence of arabinose for 48 h (Figure 2). The protein band was not found in JOL1675 grown under conditions repressing lysis gene expression. Further, morphological alteration of the JOL1675 ghosts was analyzed by SEM techniques. Transmembrane tunnels were generated on the surface of slightly elongated ghost cells, which collapsed due to the expulsion of cytoplasmic contents. These tunnels were not identified in non-lysed JOL1675 (Figure 3). These data show that expression of endolysin in JOL1675 ghosts was stringently regulated by the convergent promoter system located within the R lysis cassette, resulting in the successful inducible lysis of JOL1675.

### Lysis efficiency

**Figure 4:**
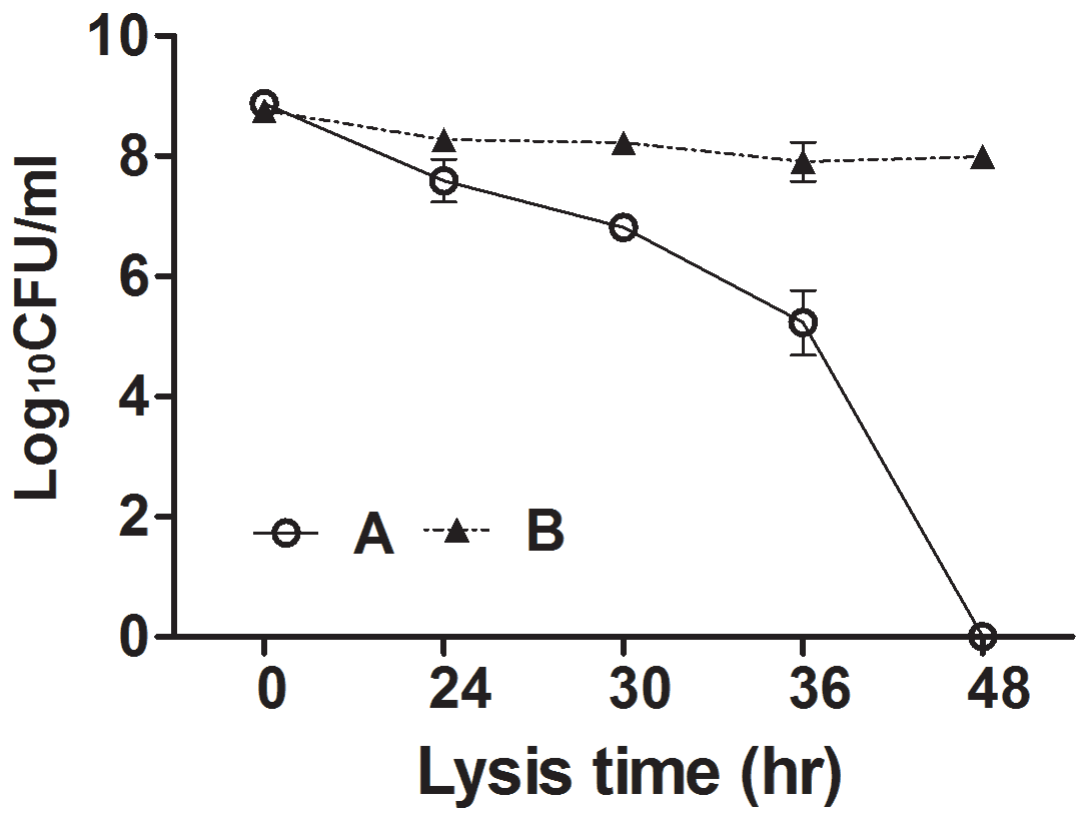
Loss of viability of JOL1675 during formation of ghost cells *S*. Typhi JOL1675 grown to exponential phase were inactivated by induction of the lysis genes. The CFU counts were transformed to log base 10 values. The data are presented as the mean ± s.d. of three samples. **(A)** JOL1675 ghost cell; **(B)** JOL1498, vector control which did not contain the ghost plasmid.

To assess the lytic capacity of the holin-endolysin component expressed in JOL1675, the decrease in the number of viable cells was monitored during ghost preparation. A JOL1675 culture containing approximately 10^9^ CFU was inactivated under conditions triggering endolysin expression. During the lysis procedure, the number of viable cells decreased progressively. After 48 h of lysis, no viable cells remained, indicating that endolysin expression occurred throughout the population of JOL1675 bacteria (Figure 4).

### Humoral and mucosal antibody responses

**Figure 5:**
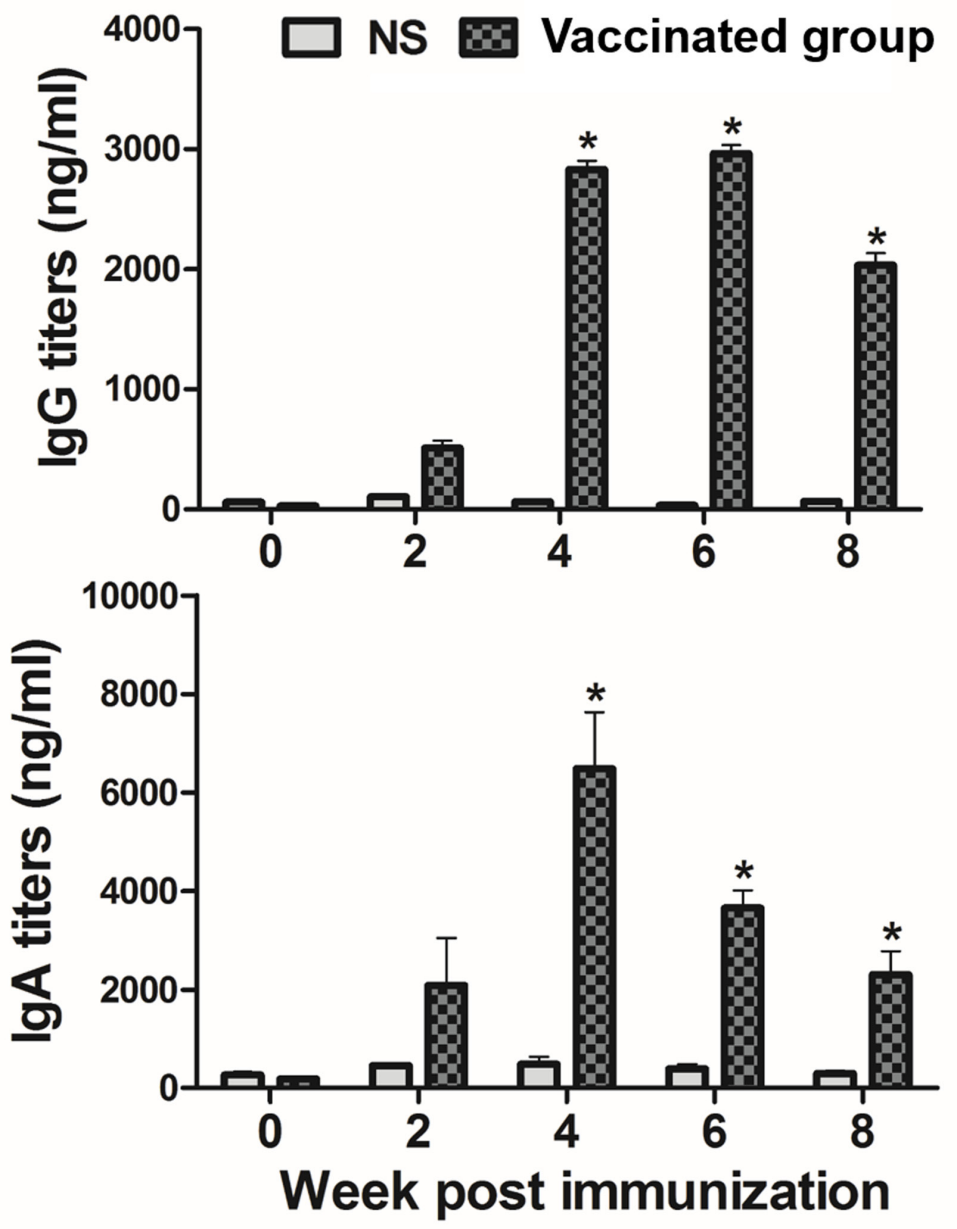
Serum IgG (I) and vaginal wash secretory IgA (II) titers against *S*. Typhi OMP in the subcutaneously immunized mice by ELISA analysis Bars indicate the mean of all mice (n=10) in each group, and the vertical lines show the s.d. NS, non-stimulated mice, * *p* < 0.05, (vs. NS).

Mice were immunized subcutaneously with JOL1675 ghosts, and serum or vaginal wash samples collected at various times after immunization were assayed, respectively, for their IgG or secretory IgA antibody (Ab) titers against*S*. Typhi outer membrane protein (OMP). The concentrations of serum IgG and vaginal wash sIgA specific to *S*. Typhi OMP were significantly increased in mice inoculated with JOL1675 ghost at weeks 4, 6, and 8 post-immunization (PI) compared to those of the non-immunized mice (*P* < 0.05) (Figure 5). IgG titer was dramatically increased at week 4 PI, two weeks after the second injection, and reached a peak response at week 6 PI. The vaginal wash sIgA titers also peaked within week 4 PI, and the significantly increased levels persisted during the observational period (*P* < 0.05).

### Serum bactericidal assay

Serum bactericidal activity (SBA) assays were performed to evaluate the capacity of functional Abs induced by JOL1675 to kill wild-type *S*. Typhi in a complement-dependent manner. Serum Abs were obtained at week 6 PI from rabbits receiving JOL1675 immunization twice, at weeks 0 and 2. SBA was determined by colony counts recovered from the reaction mixture comprised of wild-type *S*. Typhi JOL380, heat inactivated serum Abs, and exogenous complement after a 1 h incubation at 37 °C. The mixture containing serum Abs sampled from the immunized rabbits at week 6 PI showed a significantly increased bacterial killing (90.83 ± 1.02% of SBA) compared to that of the non-immunized control (2.96 ± 1.01% of SBA)(*P* < 0.05).

## DISCUSSION

In this study, we genetically engineered a *Salmonella* Typhi ghost vaccine candidate utilizing endolysin-mediated lysis event. In the infection cycle of double-stranded DNA bacteriophage λ, the holin and endolysin proteins are mainly involved with host bacterial cell lysis [14]. The holin protein, encoded by the *S* gene, permeabilizes the inner membrane (IM) of the host bacteria. At the time predetermined by holin, which controls the phage infective cycle, the muralytic enzyme endolysin, encoded by the *R* gene, accumulates in the cytoplasm and escapes through the pore of the IM to digest the peptidoglycan (PG) layer [14]. Given the capacity of holin-endolysin to hydrolyze PG and induce programmed cell death, the R lysis cassette containing *R, S, Rz*, and *Rz1* was incorporated into a novel plasmid pJHL464 for use in producing bacterial ghosts. In this study, the expression of endolysin encoded by gene *R* in JOL1675 was genetically and phenotypically confirmed by immunoblotting and SEM analysis, respectively. The expression of endolysin was revealed by detection of an immunoreactive band corresponding to the expected size of the lysis protein in Western blot analysis (Figure 2). The morphological alteration of JOL1675 ghosts, such as partial collapse, transmembrane tunnel formation, and cell elongation induced by endolysin expression, was visualized by SEM analysis (Figure 3). Further, potent lytic activity exerted by sequential transcription of the *S* and *R* genes caused production of JOL1675 ghosts within approximately 48 h after gene induction (Figure 4). Vivek *et al*. reported that an R lysis cassette employed to construct a DNA vaccine delivery vector effectively offered the steady lysis of *Salmonella* under the arabinose-inducible promoter [16]. However, this approach was limited by using it only as a DNA delivery vector but not to produce bacterial ghosts that could elicit immune responses against their own immunogenic components. During the lysis procedure, holin forms the IM lesion and endolysin destroys PG but the bacterial outer membrane does not seem to be extensively altered [17]. Rajaure *et al*. demonstrated that accessory proteins encoded by the *Rz* and *Rz*1 genes form the spanning complex that connects the IM to the OM and subsequently construct the transmembrane tunnel [18]. In this context, it is presumed that the OM of JOL1675 ghosts, which is minimally affected during ghost preparation, retains most or all of the immunogenic characteristics of the OM from viable *S*. Typhi bacteria.

**Figure 6:**
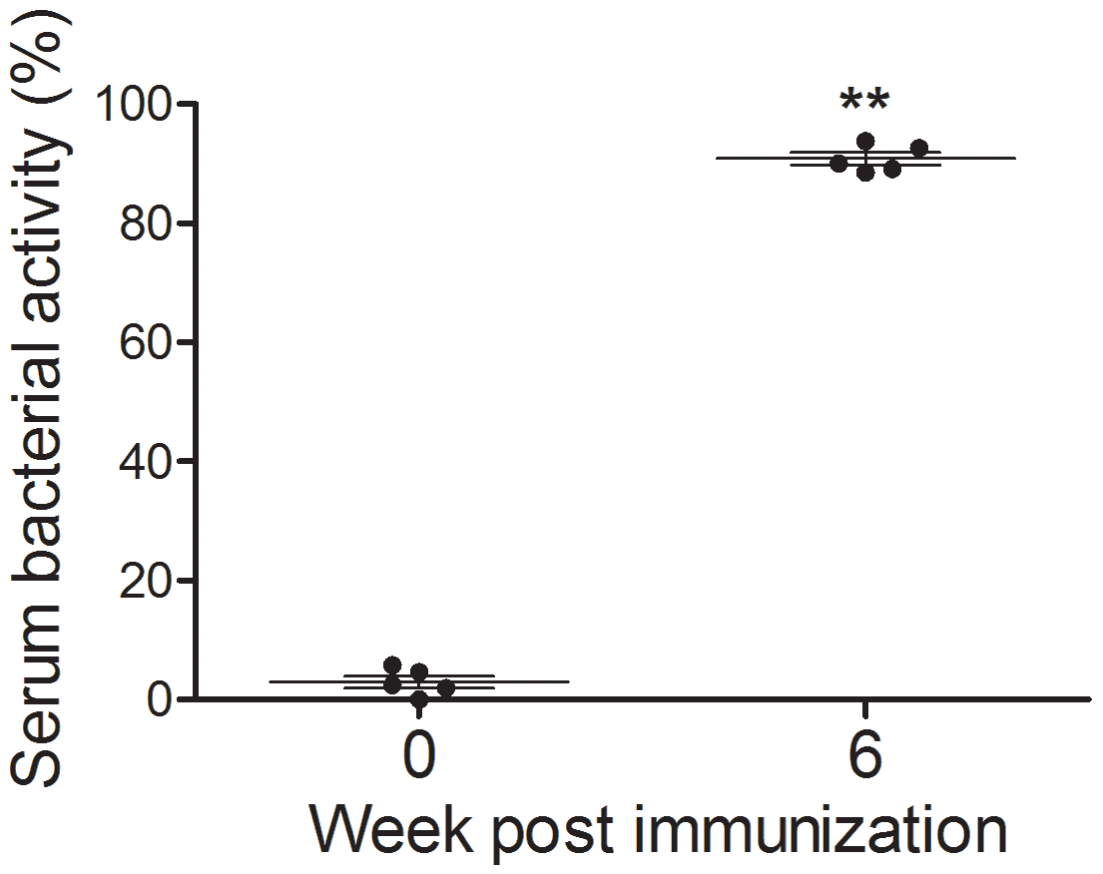
Sera were collected from rabbits immunized with JOL1675 ghost SBA was measured as the extent to which the virulent *S*. Typhi could survive in the presence of the collected serum and complement. The error bars indicate s.d. **, *p* < 0.001.

Significantly increased serum IgG and vaginal wash sIgA antibody titers specific to *S*. Typhi OMP (*P* < 0.05) were detected in mice immunized with JOL1675 ghosts in the present study (Figure 5). These results indicate that JOL1675 ghost efficiently induces humoral and mucosal immunity, which is essential for typhoid fever prevention at early stages of infection [19]. Particularly, enhanced serum IgG and intestinal IgA against the *S*. Typhi Ty21a O antigen is known as the optimal surrogate marker of protection [20]. Live attenuated *S*. Typhi Ty21a vaccine ingested via an oral route reaches the Peyer's patches, one of the primary mucosal inductive sites, and effectively induces gut-derived sIgA. In this study, markedly increased sIgA levels were also observed in the genital tract of the mice immunized with JOL1675 ghosts (*P* < 0.05) via a subcutaneous route where maximum immunogenicity was induced. Further, given a significant correlation among sIgA titers produced in various mucosal effector sites [21], the increased sIgA titers observed in this study were likely proportional to sIgA titers generated in the intestine. In addition, immunization of the rabbits with JOL1675 ghosts elicited robust complement-dependent serum bactericidal activity. The SBA assay is considered the “gold standard” to measure the ability of a vaccine to induce functional antibodies to eliminate target bacteria. Thus, the SBA assay has been used to support licensure of vaccines against bacterial diseases [22]. The serum antibodies elicited by JOL1675 ghost immunization at week 6 PI exhibited high bactericidal activity, killing approximately 91% of a virulent *S*. Typhi strain in the presence of exogenous complement (Figure 6). This markedly increased SBA activity demonstrated that antigenic determinants derived from the JOL1675 Vi-negative *S*. Typhi Ty21a [20] ghost lysed by endolysin expression have the potential to generate functional antibodies (*P* < 0.05). Purikal *et al*. previously indicated no significant correlation between SBA titer and antibodies against Vi polysaccharides [23]. Thus, they presumed that other outer membrane proteins of *S*. Typhi such as somatic O antigen and porins might be responsible for the predominant SBA activity. Collectively, outer membrane proteins preserved in JOL1675 ghosts following the lysis procedure may conserve the native structure and antigenic components of their living counterparts, which can efficiently generate functional antibodies.

In conclusion, we demonstrate the application of a novel phage λ lysis gene cassette for the production of bacterial ghosts to be used as an inactivated vaccine against *S*. Typhi infection. The holin-endolysin system successfully produced a *S*. Typhi Ty21a ghost vaccine candidate with robust immunogenicity sufficient to generate high levels of complement-dependent serum bactericidal activity *in vitro*. Considering the prominent lysis efficiency of the holin-endolysin system and the substantial immunogenicity of the resultant ghosts, the construct presented here offers a safe and accessible platform with potential to contribute greatly to development of an inactivated vaccine to prevent *S*. Typhi infection.

## MATERIALS AND METHODS

### Bacterial strains and plasmid used in this study

The bacterial strains, ghost plasmid, and primers used in this study are listed in Table 1. Strains harboring the ghost plasmid were inoculated in medium with 0.2% L-arabinose, and the *asd* gene-deleted mutants were grown at 37°C in LB broth or Brilliant Green Agar (BGA) supplemented with 50 μg/ml diaminopimelic acid (DAP). All bacterial strains and plasmid were kept at −80°C in LB broth containing 20% glycerol.

**Table 1:** Bacterial strains and plasmids used in this study

Strain/plasmids	Description	References
*E. coli*		
DH5α	*fhuA2 Δ(argF-lacZ)U169 phoA glnV44 Φ80 Δ(lacZ)M15 gyrA96 recA1 relA1 endA1 thi-1 hsdR17*	Lab stock
BL21(DE3)	139(*ara-leu*)7697 *gal*U*gal*K*rps*L (Str^r^)*end*A1 *nup*GF^-^*ompThsdSB*(rB^-^mB^-^)*dcmgalλ*(DE3) pLysSCmr	Lab stock
χ6212 (JOL232)	F-λ-φ80 Δ(*lacZYA*-*argF*) *endA1 recA1 hadR17 deoR thi-1 glnV44 gyrA96 relA1 ΔasdA4*	Lab stock
*Salmonella* Typhi		
JOL380	Wild type isolate from human	Lab stock
JOL1498	*Δasd*, Ty21a vaccine strain	Lab stock
JOL1675	JOL1498 containing pJHL464	This study
Plasmids		
pJHL319	T-easy vector harboring *E* gene ghost cassette with the convergent promoter system	This study
pJHL374	T-easy vector harboring the R ghost cassette with the convergent promoter system	This study
pJHL172	*asd*^+^vector, pBRori plasmid harboring cI857/λP_R_ promoter, *araC* P_*araBAD*_, *phi*X174 lysis gene *E*	[13]
pJHL464	*asd*^+^vector, p15Aori plasmid harboring cI857/λP_R_ promoter, *araC* P_*araBAD*_, *the R ghost cassette composed of S, R and R1/Rz genes*	This study

### Construction of the pJHL464 plasmid harboring the R lysis cassette

The R lysis cassette comprising open reading frames (ORFs) of the *S* and *R* genes and overlapping ORFs of *Rz/Rz1* genes was chemically synthesized (Bioneer, South Korea). A 1,433 bp lysis cassette fragment was digested and inserted into the NcoI/BamHI-digested pJHL319 plasmid, a T-easy vector carrying the lysis *E* gene controlled under the dual promoter system. The resultant plasmid was designated pJHL374, which contained an arabinose-inducible *araBAD* promoter and thermos-sensitive λpR promoter controlling the sequential expression of holin and endolysin. The total 4.2-kb DNA fragment that carries the R lysis cassette regulated by the convergent promoter components was subcloned via a BglII/ XhoI digested fragment and subcloned into the *asd*^+^ plasmid pJHL172 [13]. The resultant pJHL464 plasmid was initially introduced into the Δ*asd E. coli* χ6212 (JOL232) strain and was subsequently inserted into the Δ*asd S*. Typhi Ty21a JOL1498 strain by electroporation. The construct was designated as JOL1675. To purify endolysin, the *R* gene was amplified by PCR from the R lysis cassette using the following primer pair: R_F: 5’-CCGCGAATTCATGGTAGAAATCAATAATCAACG-3’ and R_R: 5’ CCGCCTCGAGTACATCAATCTCTCTGACCG-3’. The EcoRI/HindIII digested PCR product was ligated into the pET28a protein expression vector. The resulting pET28a-R plasmid was used to transform competent *E. coli* BL21 (DE3) to purify endolysin protein according to the protocol previously described [24].

### Confirmation of endolysin expression

The *in vivo* expression of endolysin in the JOL1675 ghost strain was validated by Western blot analysis using the sera of hyperimmune rabbits raised against endolysin protein as previously described [25]. The morphological alteration of JOL1675 inactivated by expression from the R lysis cassette during 48 hr was visualized by performing scanning electron microscopy (SEM) [26]. For comparison, JOL1675 grown under conditions repressing lysis gene expression was used as a negative control.

### Lysis efficiency

To evaluate the lytic capacity of endolysin expressed in JOL1675, a single colony of JOL1675 was inoculated in LB broth supplemented with 0.2% L-arabinose to mid-log phase at 27°C in a shaking incubator (80 rpm). To produce the JOL1675 ghost, the harvested culture was resuspended with LB broth and grown at 42°C with 150 rpm agitation. Lysis efficiency was measured by the viability of cells recovered in the culture collected at 12-h intervals under optimal conditions inducing expression of the R lysis cassette. Cell viability was determined by counting the colony forming units (CFUs) in 100 μl of the inoculation in triplicate. After 48 h of the lysis procedure, the genetically prepared ghost cells were collected by centrifugation (4,000×g at 4°C for 20 min), and the pellet was stored at -70°C until further processing.

### Immunization

All animal experimentation work was approved by the Chonbuk National University Animal Ethics Committee (CBNU2015-00085) and was carried out according to the guidelines of the Korean Council on Animal Care and Korean Animal Protection Law, 2007; Article 13 (Experiments with Animals). Female BALB/c mice at 5 weeks of age (n=20) were randomly assigned to two groups. The mice in group A received two intramuscular inoculations of 1 × 10^8^ of JOL1675 ghost cells at 2-week intervals. The group B mice also were intramuscularly injected with 100 μl sterile PBS for a negative control at week 0 and 2. At 0, 2, 4, 6, and 8 weeks post immunization (PI), serum was obtained from whole blood via the orbital sinus vein to measure serum IgG, and vaginal lavage fluid was sampled by washing the vagina with 200 μl of sterile PBS to detect secretory IgA [27].

### Humoral and mucosal immune responses

To assess humoral and mucosal immune responses elicited by the JOL1675 ghost, immunoglobulin (Ig) G and secretory IgA (sIgA) titers specific to *S*. Typhi outer membrane protein (OMP) were measured by enzyme-linked immunosorbent assay (ELISA) following the protocol previously described [24, 26]. ELISA microtiter plates (Greiner, Frickenhausen, Germany) were coated with OMP extracted by the wild-type *S*. Typhi JOL380 (500 μg per well). The end point titers of IgG in the mice serum samples and IgA the vaginal wash specimens were acquired by a standard curve prepared by making serial dilutions of purified mouse immunoglobulins (Southern Biotechnology, Birmingham, AL).

### Serum bactericidal assay

To evaluate the protective potential of antibodies (Abs) elicited in response to JOL1675 ghost immunization, serum bactericidal activity (SBA) assays were performed as previously described [28]. Briefly, two New Zealand white rabbits were immunized twice with 1×10^8^ JOL1675 ghosts at two-week intervals via a subcutaneous route. Serum sampled at weeks 0 and 6 PI were heat-inactivated at 56°C for 30 min to inhibit the complement alternative pathway. The inactivated serum samples were serially diluted with PBS as follows: 1:1, 1:50, and 1:100 to determine optimal serum concentrations for the assay. The wild type *S*. Typhi JOL380 was incubated to mid-logarithmic phase at 37°C in LB medium, and the cell suspension was diluted in sterile PBS to yield 10^2^ CFU per 10 μl. Baby rabbit complement (Sigma-Aldrich, St. Louis, MO) was utilized as an exogenous complement source. The mixture contained 10 μl of the optimized rabbit serum concentration (1:1) and 12.5% final concentration of baby rabbit complement, and this was incubated at 37°C for 1 h. The number of JOL380 cells surviving at the end of the procedure was measured by plating the mixture on BGA. SBA was determined using the following formula: SBA (%) = (1- (the number of colonies shown in the mixture with serum collected from the immunized group)/ (the number of colonies shown in the mixture with serum collected from the control group) × 100 [28].

### Statistical analysis

Statistical analyses were performed with GraphPad PRISM software (Intuitive Software for Science, San Diego, CA). The statistical difference between immunized and non-immunized mice was tested with independent-samples *t* test. A *p* value less than 0.05 was considered to be statistically significant.
